# Purification, Identification, and Characterization of an Endo-1,4-β-Xylanase from Wheat Malt

**DOI:** 10.3390/molecules25071572

**Published:** 2020-03-29

**Authors:** Zhaojun Peng, Yuhong Jin

**Affiliations:** College of Food Science and Engineering, Shandong Agricultural University, No. 61 Daizong Street, Tai’an 271018, China; pzj1669132682@163.com

**Keywords:** wheat malt, endo-1,4-β-xylanase, water-extractable arabinoxylan, arabinoxylan-oligosaccharides

## Abstract

In this study, an endo-1,4-β-xylanase was purified from wheat malt following the procedures of ammonium sulfate precipitation, cation-exchange chromatography, and two-step anion-exchange chromatography. The purified endo-1,4-β-xylanase had a specific activity of 3.94 u/mg, demonstrating a weight average molecular weight (Mw) of approximately 58,000 Da. After LC–MS/MS (Liquid chromatography-tandem mass spectrometry) identification, the purified enzyme had the highest matching degree with a GH10 (Glycoside Hydrolase 10) domain-containing protein from wheat, there were 23 match peptides with a score above the threshold and the prot-cover was 45.5%. The resulting purified enzyme was used to investigate its degradation ability on high viscosity wheat-derived water-extractable arabinoxylan (WEAX). Degradation experiments confirmed that the purified enzyme was a true endo-acting enzyme, which could degrade large WEAX into smaller WEAX. The average degree of polymerization (avDP) and the viscosity of WEAX decreased with the increasing reaction time. The enzyme could degrade a small amount of WEAX into arabinoxylan-oligosaccharides (AXOS) with a degree of polymerization of 2–6, but no monosaccharide was produced. The degradation occurred rapidly in the first 3.5 h and decreased with the further prolongation of reaction time.

## 1. Introduction

Nowadays, wheat beer is gaining popularity in China, and even in other countries of the world, because of its mellow taste and rich and lasting foam. At least 40% of wheat malts are used in the production of wheat beer. Correspondingly, the demand for wheat malt is also increasing due to the prevalence of wheat beer in recent years [1].

Unlike barley beer, the presence of a high amount of macromolecule polysaccharide in wheat can decrease the beer filtration rate [2], increasing the production cost of beer. Arabinoxylan (AX) is one of the main components in the endosperm cell walls of wheat [3], which has a linear backbone of (1,4)-β-D-xylopyranose with α-L-arabinofuranose substitutions [4]. Arabinoxylan can be classified into water-soluble arabinoxylan (WEAX) and water-insoluble arabinoxylan (WUAX) based on their solubility [5]. The content of AX in wheat is about 3–12.5% [6]. The content of WEAX in wheat malt is 1.08% ~ 1.16% [7], which is much higher than that in barley malt.

Xylanase is a series of endogenous enzymes that is present in wheat malt, including endo-1,4-β-xylanase, arabinofuranosidase, β-D-xylosidase, feruloyl esterase, and so on; among these, endo-1,4-β-xylanase is the key enzyme for the degradation of AX, which breaks the β-(1,4)-glycosidic bond between xyloses (Xyls) [8], reduces the polymerization degree of AX, and changes the quantity and distribution of the arabinose (Ara) residues, which further alters the physical and chemical properties of AX dramatically.

The endogenous endo-1,4-β-xylanase plays an important role in regulating the content and molecular size of WEAX in wheat malting and wheat beer production. In the wheat malting and wort mashing process, WUAX is degraded to WEAX, and WEAX is further degraded by endogenous endo-1,4-β-xylanase, which affects the viscosity, turbidity, and filtration speed of wort and beer. Water-soluble arabinoxylan with a high Mw at a high concentration increases the viscosity of the wort [9] and reduces the filtration rate [10]. In contrast, WEAX with a medium Mw is beneficial to the foam stability and mellowness, which improves the taste and flavor of beer [11]. From the perspective of nutrition, WEAX with small Mw, particularly for xylooligosaccharide, was a good prebiotic, which also exhibits great laxative and anti-tumor effects [12].

Different types of wheat beer, such as cloudy wheat beer and clarified wheat beer, have different requirements in terms of viscosity and turbidity and therefore different requirements for AX content during wheat malt and brewing technology. Firstly, it should be determined exactly how much of a role the endogenous endo-1,4-β-xylanase plays in the process of wheat malting and wort preparation, such as its degradation efficiency on WEAX and WUAX, molecular size, the viscosity, and turbidity characteristics of degradation products, etc. Further, it should be guaranteed that AX degradation in wheat malting and wort mashing can be freely and reasonably controlled. However, there are few reports in this field. Endo-1,4-β-xylanase is a highly diversified enzyme [13] in various aspects such as structural domains, biochemical properties and catalytic characteristics. Many studies have been carried out to improve the enzyme stability through genetic recombination [14] and modification. Previous researchers have investigated the effect of endo-1,4-β-xylanase on the degradation of the arabinoxylan present in barley and noticed the release of oligomers in dehusked barley grain [15,16]. Cleemput et al. [17,18] identified two different endo-1,4-β-xylanases in wheat flour, whereas the Mw, isoelectric point, substrate, and product specificity varied dramatically [19].

In this study, we wanted to separate endo-1,4-β-xylanase from malt and study its enzymatic properties, degradation mechanism and degradation efficiency on WUAX and WEAX. The results of this study will guide the reasonable degradation of AX during the production of wheat malts and wheat beer. Finally, the purified endo-1,4-β-xylanase will be used in the production of wheat beer to assist in the degradation of AX in wheat malt and wort. In this study, a novel endo-1,4-β-xylanase was purified from wheat malt, and proteomics identification and partial degradation characteristics on high-viscosity wheat-derived WEAX were verified. The in-depth study of further degradation characteristics of the purified enzyme on WUAX and WEAX and its application in the beer industry are being studied.

## 2. Results and Discussion

### 2.1. Wheat Malt Endo-1,4-β-Xylanase

#### 2.1.1. Wheat Malt Endo-1,4-β-Xylanase Purification

As shown in Table 1, the activity of crude enzyme extracted from wheat malt were 3538 u and the amount of protein was 8231 mg. The corresponding enzyme activities for AS0-20 (0–20% ammonium sulfate precipitated protein), AS20-40, AS40-60, AS60-80, and AS80-100 were 55, 125, 30, 3, and 19 u, respectively. Therefore, the two precipitates (AS0-20 and AS20-40) with higher specific activities (0.06 u/mg and 0.10 u/mg) were used for further purification.

**Figure 1:**
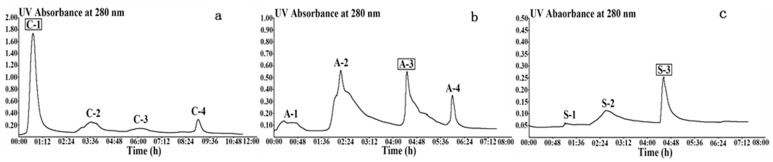
Chromatography of purification of endo-1,4-β-xylanase. (**a**) SP-Sepharose Fast Flow cation exchange chromatography. (**b**) Q-Sepharose Fast Flow anion-exchange chromatography. (**c**) Source 30Q anion-exchange chromatography. The ordinate of (**a**–**c**) is absorbance (280 nm) and the abscissa is time (h).

Figure 1a shows the elution profiles of AS0-20 and AS20-40, which were separated using SP-Sepharose Fast Flow cation exchange chromatography. Four peaks (C-1, C-2, C-3, and C-4) were separated when phosphate buffers containing different concentrations (0, 0.1, 0.2, and 0.5 M) of NaCl were used as elution buffer. The C-1 peak had the highest endo-1,4-β-xylanase activity of 649 u, and the amount of protein was 1405 mg. The corresponding specific activity was 0.46 u/mg and purification fold was 1.0.

The collected enzyme solution from peak C-1 was further purified by Q-Sepharose Fast Flow anion exchange chromatography. As shown in Figure 1b, four peaks (A-1, A-2, A-3, and A-4) were detected after eluting with 0.05 M Tris-HCl buffer (pH 8.3, Buffer II) containing different concentrations of NaCl: 0, 0.1, 0.2, 0.3 M. Peak A-3 exhibited the highest specific activity of 2.09 u/mg. Compared to C-1, the total activities of arabinofuranosidase and β-d-xylosidase for A-3 decreased significantly from 2588 u and 12043 u to 513 u and 2466 u, respectively.

Source 30Q anion exchange chromatography was further used to purify peak A-3, and the results are shown in Figure 1c. The enzyme solution corresponding to three peaks (S-1, S-2, S-3) was eluted by 0.1, 0.2, 0.3 M NaCl (Buffer II) and dialyzed, to measure the endo-1,4-β-xylanase activity and the amount of protein. As demonstrated in Table 1, peak S-3 had the highest specific activity of 3.94 u/mg. The purification fold of the endo-1,4-β-xylanase was 9.0 and the recovery rate was 0.7%. Moreover, it was noticed that the activities of arabinofuranosidase and β-d-xylosidase were 0.27 u and 1.47 u, respectively. Therefore, peak S-3 was chosen as the final product to degrade the high-viscosity WEAX. Compared to the previously separated endo-1,4-β-xylanases [17,18,20], the enzyme obtained in this study had a relatively high endoxylanase activity and almost no exoxylanase activity. 

**Figure 2:**
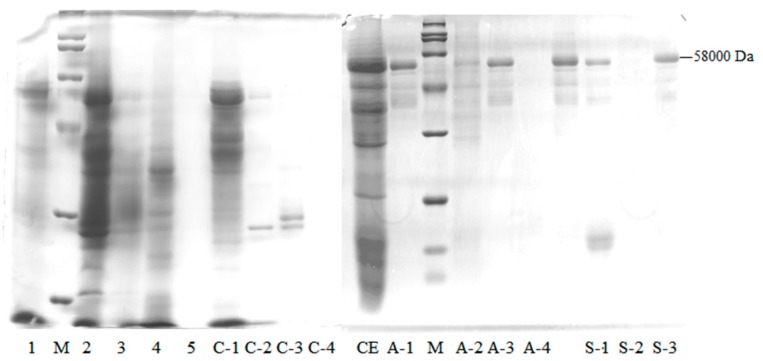
SDS-PAGE of the endo-1,4-β-xylanase. Lane CE represent crude enzyme; Lanes 1, 2, 3, 4, and 5 represent AS0-20, AS20-40, AS40-60, AS60-80, and AS80-100, respectively; Lanes C-1, 2, 3, 4 represent the four peaks eluted by SP-Sepharose Fast Flow cation chromatography, respectively; Lanes A-1, 2, 3, 4 represent the four peaks eluted by Q-Sepharose Fast Flow anion chromatography, respectively; Lanes S-1, 2, 3 represent the three peaks eluted by Source 30Q anion-exchange chromatography, respectively; M is the protein marker. Note: from top to bottom, the molecular weights of protein markers are 200, 116, 97.2, 66.4, 44.3, 29.0, 20.1, 14.3, and 6.5 kDa.

Figure 2 exhibits the sodium dodecyl sulfate polyacrylamide gel electrophoresis (SDS-PAGE) electrophoretogram of enzyme solution after different purification steps. Comparing the bands of A-2, S-1, S-3 with the activities of endo-1,4-β-xylanase, it could be inferred that the Mw of the target band in S-3 was about 58,000 Da. This was close to the Mw of endo-xylanase (55,000 Da) in wheat flour purified by Cleemput et al. [17].

#### 2.1.2. LC–MS/MS Proteomic Identification of a Purified Enzyme

The proteomic identification of the purified enzyme with the Mw of approximately 58,000 Da as shown in SDS-PAGE electrophoresis (Figure 2) was determined using LC–MS/MS. The matched proteins with a prot-score ≥ 80 were selected, and their information was listed in Table 2. As shown, the purified enzyme had a high matching degree with endo-1,4-β-xylanase from barley, including Q94G07, P93185 and P93187.

Among all matched proteins, A0A3B6MY89, A0A3B6LV77, A0A3B6KNQ8 from wheat had the highest matching degree. All three proteins contained the same domain of the Glycoside Hydrolase Family 10, which was in agreement with previous research, where it was reported that endo-1,4-β-xylanase belonged to the GH10 and GH11 families [21]. Based on the UniProt database blast result, it was found that there were 69, 66, and 48 proteins named endo-1,4-β-xylanase homologous to A0A3B6MY89, A0A3B6LV77, A0A3B6KNQ8, respectively. Blast results also showed that A0A3B6MY89 and A0A3B6LV77 had high homology with endo-1,4-β-xylanase derived from wheat (*Triticum aestivum*) and barley (*Hordeum vulgare*) while A0A3B6KNQ8 had high homology with Triticum (*Triticum urartu*) derived from urartu. For example, as shown in Table 3, A0A3B6MY89 is homologous to protein Q9XGT8, the name of which is (1,4)-beta-xylan endohydrolase (*Triticum aestivum*) and the identity is 94.80%. A0A3B6MY89 is also homologous to protein P93185, which is a (1,4)-beta-xylan endohydrolase, isoenzyme X-I from barley (*Hordeum vulgare*) and the identity is 90.70%. Thus, the three proteins (A0A3B6MY89, A0A3B6LV77, and A0A3B6KNQ8) that contain GH10 domain in Table 2 should be endo-1,4-β-xylanase.

A0A3B6MY89 had the highest matching degree with the purified endo-1,4-β-xylanase, which had a prot-score of 586, prot-mass 60827 Da and a sequence with 547 amino acids. In total, there were 30 matched peptides of A0A3B6MY89 with purified endo-1,4-β-xylanase, including 23 match peptides with a score above the threshold. The prot-cover, which was the ratio of the number of matched amino acids to the total number of amino acids of A0A3B6MY89, was 45.5%. The matched amino acids sequences are summarized in Table 4.

### 2.2. Degradation Effect of the Purified Endo-1,4-β-Xylanase on Wheat-Derived WEAX

#### 2.2.1. Changes of WEAX Content, avDP, and AXOS Content

Table 4 summarizes the changes of average degree of polymerization (avDP), content of WEAX, and content of arabinoxylan-oligosaccharides (AXOS) as a function of enzymatic hydrolysis time. The avDP of WEAX decreased from 25.29 to 16.22 after degradation for 3.5 h to 24 h, indicating that the length of WEAX chain became shorter under the action of the purified endo-1,4-β-xylanase.

Moreover, it can be seen from Table 5 that the WEAX content decreased with the increasing reaction time along with the formation of AXOS, with an avDP of 2–6. The increasing reaction time increased the concentrations of AXOS (AXOS2-AXOS6). However, after degradation for more than 3.5 h, only a slight increase of AXOS2 was detected, while others increased rapidly. The results were in agreement with the research of Cleemput et al. [18], who used arabinoxylanase extracted from wheat flour to degrade arabinoxylans and detected, apart from Ara and Xyl, high levels of AXOS with a degree of polymerization of two to five, along with some unidentified products. It should be noted that no free monosaccharides (Ara and Xyl) were detected during the degradation in this study, indicating that the purified enzyme was a true endo-acting enzyme and could degrade a small amount of WEAX into AXOS. Similar results were also noticed when purified endo-xylanase from *Trichoderma inhamatum* was used to degrade oat spelt xylan; the enzymes released xylobiose and larger xylooligosaccharides and were classified as endoxylanases [22].

#### 2.2.2. Changes of WEAX Mw and Viscosity

**Figure 3:**
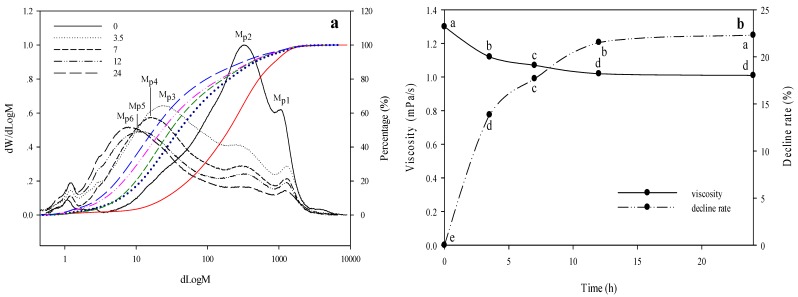
High-performance gel filtration chromatography and the change of viscosity of high-viscosity wheat-derived WEAX after the degradation by endo-1,4-β-xylanase. (**a**) Mw changes of WEAX. (**b**) viscosity changes of WEAX.

The Mw and viscosity of WEAX before and after degradation were summarized in Figure 3. As indicated in Figure 3a, the addition of the enzyme significantly shifted the peak (Mp) of WEAX to a lower Mw direction, indicating the strong degradation effect of the enzyme on WEAX. After reacting for 3.5 h, the Mw decreased rapidly from 34.60 × 10^4^ to 17.36 × 10^4^ Da. A further increase of the reaction time resulted in a slight shift of the peak. With the prolongation of degradation time, the main peak Mw of WEAX decreased in turn from 31.71 × 10^4^ (M_p2_) to 2.30 × 10^4^ (M_p3_), 1.56 × 10^4^ (M_p4_), 1.21 × 10^4^ (M_p5_), and 0.83 × 10^4^ (M_p6_) Da. In short, as the degradation time increased, WEAX could be degraded by the endo-1,4-β-xylanase to a smaller Mw WEAX.

Figure 3b shows the changes of the viscosity of WEAX as a function of reaction time. The WEAX solution had a viscosity value of 1.30 ± 0.00 mPa·s. The enzymatic reaction reduced the viscosity to 1.12 ± 0.00 mPa·s at 3.5 h. The decreased viscosity was apparently a result of the decreased molecular weight of WEAX (Figure 3a). A further increase of reaction time only slightly decreased the viscosity, which was in agreement with the changes of molecular weight shown in Figure 3a. All the results showed that the purified endo-1,4-β-xylanase had an obvious degradation effect on WEAX.

#### 2.2.3. Changes of WEAX Surface Morphological

**Figure 4:**
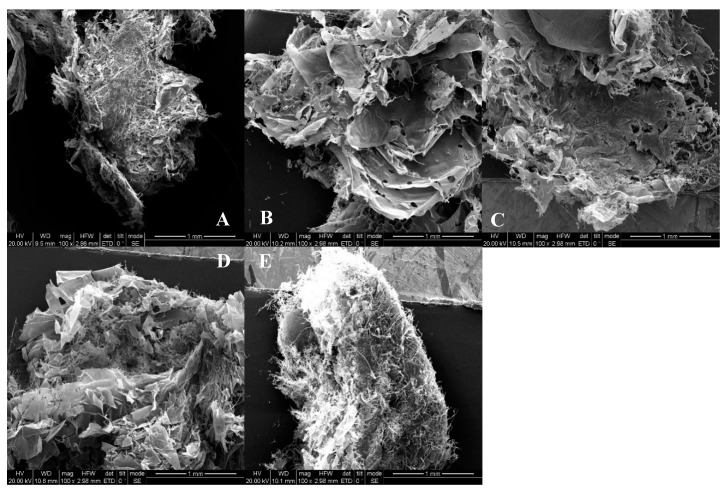
Surface morphological images of WEAX before and after the degradation by endo-1,4-β-xylanase. Note: the five pictures (**A**–**E**) represent the surface morphological images of WEAX after degradation for 0, 3.5, 7, 12, and 24 h, respectively.

The SEM pictures of the WEAX before and after being enzymatically hydrolysized are listed in Figure 4. Figure 4A showed that WEAX without enzymatic treatment had an irregular shape and a compact structure, with some flocculent substances on the surface. A few holes were observed (Figure 4B) and the WEAX had a loose, irregular sheet shape. After 7 h of degradation, the WEAX showed fragmentation (Figure 4C). After 12 h of degradation (Figure 4D), the flake shape of WEAX disappeared and the structure became fluffier. When degraded for 24 h (Figure 4E), the WEAX was compact, with many filaments surrounding the surface. The above results indicated that the purified endo-1,4-β-xylanase could not only affect the main chain of wheat WEAX but also modify its surface morphology. In other words, the endo-1,4-β-xylanase could degrade WEAX, resulting in the formation of smaller molecules and more fragmented flocculent surface structures.

## 3. Materials and Methods

### 3.1. Experimental Materials

Preparation of sterile wheat malt: the germination parameters were from the method of Xie et al. [23]. The soaking water was sterile water which was prepared from water filtered by a 0.45 μm membrane, and 0.13% H_2_O_2_ was added to the soaking water when the raw wheat was first soaked. The air used for ventilation during germination passed through a sterile filter. Ammonium sulfate and substrate (4-*O*-Methyl-d-xylan dyed with Remazol Brilliant Blue R) for determining the endo-1,4-β-xylanase activity were purchased from Sigma (St. Louis, LA, USA); p-nitrophenyl-α-l-arabinofuranoside and p-nitrophenyl-β-d-xyloside were purchased from Zibo Feiyang Biotechnology Co., Ltd. (Zibo, China). WEAX (Product code: P-WAXYH; Purity: 95%; viscosity: 42 cSt; Arabinose: Xylose (A/X) = 38/62) derived from wheat was ordered from Megazyme International (Ireland). Xylobiose, xylotriose, xylotetraose, xylopentose, xylohexasaccharide, and dialysis tubes (MWCO 3,500 Da) were obtained from Shanghai Yuanye Biotechnology Co., Ltd. (Shanghai, China). Ara, Xyl, Mannose, Galactose, and glucose were purchased from Sigma (St. Louis, LA, USA). P-82 pullulan (Denko KK, Tokyo, Japan) with different Mws (80.5 × 10^4^, 34.8 × 10^4^, 20.0 × 10^4^, 11.3 × 10^4^, 4.88 × 10^4^, 2.17 × 10^4^, 1.00 × 10^4^, 0.62 × 10^4^, 0.132 × 10^4^, and 0.342 × 10^3^ Da) were used as the standards. All other reagents used in this study were of at least analytical grade.

### 3.2. Purification of the Wheat Malt Endo-1,4-β-Xylanase

All purification steps were carried out under sterile conditions.

#### 3.2.1. Crude Enzyme Extraction and Fractional Ammonium Sulfate Precipitation

The crude enzyme extraction and ammonium sulfate precipitation for the crude enzyme were performed according to the method of Guo et al. [24]. The ammonium sulfate precipitated proteins were collected and labeled as AS0-20, AS20-40, AS40-60, AS60-80, and AS80-100, respectively. Then, these precipitates were dissolved in 0.05 M phosphate buffer (pH 5.5, Buffer I), respectively, and dialyzed against the same buffer at 4 °C for 24 h. The dialysate was used to measure the enzyme activities and protein contents. The sample with the highest specific activity was used for the next step.

#### 3.2.2. SP-Sepharose Fast Flow Cation Exchange Chromatography

The selected enzyme solution with the highest specific activity was centrifuged at 6000 *g* at 4 °C for 15 min and filtered by a 0.45 μm filter membrane before loading onto a SP-Sepharose Fast Flow column preequilibrated (General Electric Company, Boston, MA, USA) with 1000 mL of Buffer I. Proteins were eluted by gradient elution with a series of 500 mL of Buffer I containing sodium chloride concentration of 0, 0.1, 0.2, and 0.5 M, respectively, at a flow rate of 4.0 mL/min. A UV detector (Shanghai Qingpu Huxi Instrument Factory, Shanghai City, China) was used to monitor the protein absorbance at 280 nm. Subsequently, the enzyme activities and protein contents were determined, and the eluent with the highest specific activity was collected for the following experiment.

#### 3.2.3. Q-Sepharose Fast Flow Anion Exchange Chromatography

The selected enzyme solution was dialyzed against 0.05 M phosphate buffer (pH 7.0) and Buffer II, respectively, at 4 °C for 24 h before further fractionation using Q-Sepharose Fast Flow anion exchange chromatography (Beijing Ruida Henghui Technology Development Co., Ltd, Beijing City, China). Before loading onto the anion column equilibrated with 1000 mL of Buffer II, the enzyme solution was filtered through a 0.45 μm membrane filter to remove the big particles. The enzymes were eluted by a gradient procedure with a series of 500 mL of Buffer II containing sodium chloride concentrations of 0, 0.1, 0.2, 0.3 M, respectively. The elution was performed at a flow rate of 5.0 mL/min. The gradient enzyme solution with the highest specific activity was used for the next step.

#### 3.2.4. Source 30Q Anion Exchange Chromatography

The selected enzyme solution was dialyzed against Buffer II at 4 °C for 12 h. A Source 30Q column pre-equilibrated with 300 mL of Buffer II was used for further purification. In this case, a gradient procedure was performed with Buffer II containing 0, 0.1, 0.2, 0.3 M of NaCl at a flow rate of 1.0 mL/min. The sample with the highest specific activity was used to the further research.

### 3.3. Analysis Techniques

#### 3.3.1. Measurement of Endo-1,4-β-Xylanase Activity

The endo-1,4-β-xylanase activity was determined according to the method reported by Peng et al. [25]. A total of 1 µmol substrate (4-*O*-Methyl-d-xylan dyed with Remazol Brilliant Blue R) is converted in 1 min per g dry wheat malt is defined as an endo-1,4-β-xylanase activity unit (u).

#### 3.3.2. β-D-Xylosidase Activity Assay

The β-d-xylosidase activity was determined using the method reported in a previous study [26].

#### 3.3.3. Arabinofuranosidase Activity Assay

The arabinofuranosidase activity was determined using p-nitrophenyl-α-l-arabinofuranoside as the substrate according to the method reported previously [27].

#### 3.3.4. Protein Content Determination

Protein content, purification fold and recovery rate were determined according to the method by Guo et al. [24]. The protein standard curve was fitted to Equation Y = 0.0464X + 0.0019 with R^2^ of 0.9998, where X is the protein content (mg), Y is the absorbance at 540 nm.

#### 3.3.5. SDS-PAGE

The Mw of purified enzyme was determined by SDS-PAGE (Beijing Baijing Biotechnology Co., Ltd, Beijing City, China) on polyacrylamide gels according to the method of Guo et al. [24].

#### 3.3.6. LC–MS/MS of Purified Enzyme

For the LC–MS/MS (nanoLC-Ultra 1D Plus and TripleTOF 5600, both are produced by AB SCIEX, Waltham, MA, America) sample processing steps of the target protein, refer to Kong et al. [28]. Protein identification was performed with MASCOT software (2.5.1 Version, Matrix Science, Columbia, MO, USA) (http://www.matrixscience.com/) by searching the uniprot database. The database used for the search was a self-built database including “xylan”, “xylanase” and “GH10 domain containing protein” from wheat and barley. The searching parameters were as Table 6:

### 3.4. Degradation Ability on Wheat-Derived Arabinoxylans

#### 3.4.1. Degradation Method

The enzyme degradation process was carried out in five 250 mL flasks. Five flasks were charged with 12.5 mL enzyme solution (dissolved in Buffer I) and 77.5 mL 1.16 mg/mL water-soluble wheat-derived high-viscosity arabinoxylan solution (dissolved in water), respectively. The reaction was carried out in a shaking water bath at 40 °C. When the reaction time was 0, 3.5, 7, 12, and 24 h, a trig flask was removed, and the reaction solution inside was boiled for 15 min to terminate the reaction, and then cooled down to room temperature. Boiled solution was centrifuged at 10,000× *g* for 10 min. The supernatant was collected and filtered through a 0.45 μm filter to obtain enzymatic hydrolysate for the following experiments.

#### 3.4.2. Arabinoxylan-Oligosaccharides (AXOS) Content Determination

The contents of AXOS—the degree of polymerization of which was 2–6—were determined using Hi-Plex Na column (300 mm × 7.7 mm i.d.) (Agilent, California, CA, USA) on a high-performance liquid chromatography system (Shimadzu) coupled with a refractive index detector (RID-10). Degraded samples were filtered through the 0.45 μm and 0.22 μm membrane filters (Millipore Co., Milford, MA, USA). The injection volume of the samples was 10 μL. Elution was carried out at a flow rate of 0.2 mL/min at 80 °C with water as the mobile phase. Xylobiose, Xylotriose, Xylotetraose, Xylopentose, Xylohexasaccharide were used as the standards, and the corresponding AXOS were labeled as AXOS2, AXOS3, AXOS4, AXOS5, AXOS6.

#### 3.4.3. The Content and avDP Analysis of WEAX

The average degree of polymerization (avDP) and WEAX content were determined by gas chromatography, as described in previous reports [29,30,31,32]. Moreover, in order to detect if there were free monosaccharides in the samples, partial samples were not hydrolyzed but reduced directly with NaBH_4_ and acetylated with acetic acid anhydride.

The avDP and WEAX contents of the enzymatic hydrolysate were calculated using the Formula (1) and (2):

avDP = [%Ara + %Xyl − %(Ara + Xyl) in AXOS] / (% reducing end Xyl − % reducing end Xyl in AXOS)(1)

WEAX = [%Ara + %Xyl − %(Ara + Xyl) in AXOS] × 0.88(2)

Note: % present concentration; %(Ara + Xyl) in AXOS = %(Ara + Xyl) in AXOS2 + %(Ara + Xyl) in AXOS3 + … + %(Ara + Xyl) in AXOS6; %(Ara + Xyl) in AXOSn = % AXOSn + (n − 1) × 18 × %AXOSn / molar mass of AXOSn (n = 2, 3, 4, 5, 6); % reducing end Xyl in AXOS = % reducing end Xyl in AXOS2 + % reducing end Xyl in AXOS3 + … + % reducing end Xyl in AXOS6; % reducing end Xyl in Xn = % AXOSn / molar mass of AXOSn × molar mass of Xyl (n = 2, 3, 4, 5, 6).

#### 3.4.4. Determination of the Mw

The Mw of WEAX was determined according to the method of Jiang et al. [33].

#### 3.4.5. Viscosity Determination

The viscosity of the liquid was measured by the capillary method with the Ostwald viscosimeter (Taizhou Jiaojiang Glass Instrument Factory, Taizhou City, Zhejiang Province, China) according to the method of Agustín et al. [34].

#### 3.4.6. Scanning Electron Microscopy (SEM)

Ethanol was slowly added into five enzymatic hydrolysates with constant stirring at room temperature to a final concentration of 80% (*w*/*v*). The resulting solution was incubated at 4 °C overnight to facilitate the aggregation. Then, the precipitates were collected by centrifugation for 15 min at 6000× *g* and lyophilized to dry powders.

The SEM analysis of powder products after degradation was performed following the method of Jiang et al. [33].

### 3.5. Statistical Analysis

Excel 2017 and IBM SPSS Statistics 22 were used for the data analysis. Differentiation analysis was carried out by DPS 7.05. Different letters indicated the significant differences between two points (*p* < 0.05). All data were the mean values of at least three parallel experiments.

## 4. Conclusions

In this study, an endo-1,4-β-xylanase with a Mw of approximately 58,000 Da was isolated from wheat malt and purified more than nine-fold by ammonium sulfate precipitation, and cation- and two-step anion-exchange chromatography. The purified enzyme had the highest matching degree with a GH10 domain-containing protein from wheat, which had a prot-mass 60827 Da and a sequence with 547 amino acids; there were 23 match peptides and the prot-cover was 45.5%. The purified enzyme was a true endo-acting enzyme which could hydrolyze high-viscosity wheat WEAX into smaller molecules and also degrade a small amount of WEAXs to AXOS (with a degree of polymerization of 2–6) and no monosaccharide was found in the reaction system. The degradation occurred rapidly in the first 3.5 h.

## Figures and Tables

**Table 1:** Purification of endo-1,4-β-xylanase from wheat malt.

Fraction	Total Protein (mg)	Endo-1,4-β-Xylanase Activity (u)	Specific Activity (u/mg)	Purification Fold	Recovery Rate (%)	Arabinofuranosidase Activity (u)	β-d-xylosidase Activity (u)
Crude enzyme	8231 ± 286.18	3538 ± 106.33	0.43 ± 0.02		100	23445 ± 497.00	131825 ± 70.76
AS0-20	857 ± 4.23	55 ± 0.80	0.06 ± 0.00	0.1	2	129 ± 2.74	877 ± 2.74
AS20-40	1270 ± 11.06	125 ± 0.78	0.10 ± 0.00	0.2	4	2588 ± 47.74	12043 ± 0.00
C-1	1405 ± 42.38	649 ± 11.47	0.46 ± 0.01	1	18	513 ± 8.55	2466 ± 75.62
A-3	234 ± 12.93	490 ± 0.00	2.09 ± 0.12	5	14	36 ± 0.00	160 ± 24.25
S-3	6.08 ± 0.00	23.95 ± 1.33	3.94 ± 0.22	9	0.7	0.27 ± 0.07	1.47 ± 0.23

Notes: AS0-20 is 0–20% ammonium sulfate precipitated protein; AS20-40 is 20–40% ammonium sulfate precipitated protein; C-1 is a peak of the highest enzyme activity eluted by cation chromatography (SP-Sepharose Fast Flow); A-3 is a peak of the highest enzyme activity eluted by anion chromatography (Q-Sepharose Fast Flow); S-3 is a peak of the highest enzyme activity eluted by anion chromatography (Source 30Q).

**Table 2:** The matched protein information of LC–MS/MS.

Num	Prot_Access	Prot_Describe	Prot-Score	Prot-Mass	Matches_Sig	Sequences_Sig	Prot-Cover	emPAI
1	tr|A0A3B6MY89|A0A3B6MY89_WHEAT	**GH10 domain-containing protein** OS=*Triticum aestivum* OX=4565 PE=4 SV=1	585	60827	26	23	45.5	2.36
2	tr|A0A3B6LV77|A0A3B6LV77_WHEAT	**GH10 domain-containing protein** OS=*Triticum aestivum* OX=4565 PE=4 SV=1	465	61183	21	18	41.5	1.57
3	tr|A0A3B6KNQ8|A0A3B6KNQ8_WHEAT	**GH10 domain-containing protein** OS=*Triticum aestivum* OX=4565 PE=4 SV=1	437	61298	21	19	43.2	1.84
4	tr|Q9ZR33|Q9ZR33_WHEAT	Glycosyltransferase 75 OS=*Triticum aestivum* OX=4565 GN=rgp PE=2 SV=1	153	41985	6	6	43.2	0.58
5	tr|O04869|O04869_HORVU	Phenylalanine ammonia-lyase (Fragment) OS=*Hordeum vulgare* OX=4513 GN=PAL PE=2 SV=1	135	54553	5	5	23.5	0.34
6	tr|W5C539|W5C539_WHEAT	GT75-3 OS=*Triticum aestivum* OX=4565 PE=2 SV=1	132	41291	5	5	23.9	0.47
7	tr|A0A3B6LUY6|A0A3B6LUY6_WHEAT	GH10 domain-containing protein OS=*Triticum aestivum* OX=4565 PE=4 SV=1	112	64488	7	6	15.5	0.35
9	tr|Q94G07|Q94G07_HORVU	1,4-beta-D xylan xylanohydrolase (Fragment) OS=*Hordeum vulgare* OX=4513 PE=2 SV=1	103	55951	8	8	20.7	0.58
8	tr|P93185|P93185_HORVU	(1,4)-beta-xylan endohydrolase, isoenzyme X-I OS=*Hordeum vulgare* OX=4513 PE=2 SV=1	103	48388	8	8	21.5	0.69
10	tr|A0A3B6KR49|A0A3B6KR49_WHEAT	GH10 domain-containing protein OS=*Triticum aestivum* OX=4565 PE=4 SV=1	100	60806	7	7	16.8	0.45
11	tr|P93187|P93187_HORVU	Xylan endohydrolase isoenzyme X-I OS=*Hordeum vulgare* OX=4513 PE=4 SV=1	100	48273	7	7	22.7	0.59
12	tr|A0A3B6LTQ3|A0A3B6LTQ3_WHEAT	GH10 domain-containing protein OS=*Triticum aestivum* OX=4565 PE=4 SV=1	80	61306	6	5	14.9	0.3

**Table 3:** Similar proteins Information of A0A3B6MY89 in the UniProt database.

Num	Entry	Protein Names	Identity
1	A0A061FEZ9	Endo-1,4-beta-xylanase Z (*Aegilops tauschii*)	98.70%
2	Q9XGT8	(1,4)-beta-xylan endohydrolase (*Triticum aestivum*)	94.80%
3	P93187	Xylan endohydrolase isoenzyme X-I (*Hordeum vulgare*)	90.70%
4	P93185	(1,4)-beta-xylan endohydrolase, isoenzyme X-I (*Hordeum vulgare*)	90.70%
5	Q94G07	1,4-beta-d xylan xylanohydrolase (*Hordeum vulgare*)	89.00%
6	Q4H019	Endo-1,4-beta-xylanase (*Hordeum vulgare*)	87.90%
7	Q94G06	1,4-beta-d xylan xylanohydrolase (*Hordeum vulgare*)	87.40%
8	Q94G05	1,4-beta-d xylan xylanohydrolase (*Hordeum vulgare*)	87.00%

**Table 4:** The matched amino acid sequences between purified endo-1,4-β-xylanase and A0A3B6MY89.

Num	Sequence	Num	Sequence	Num	Sequence
1	GAVVGGIGLQGHVQNPVGEVIGAAIDRLAK	11	DGVRLPIPVGVLKPGITYR	21	GNVDGDGDFKFR
2	GAVVGGIGLQGHVQNPVGEVIGAAIDR	12	GHCVFWSVDGDVQQWVK	22	HYDVNNEMLHGR
3	LGDEDIPAYMFK	13	LRTEPR	23	AKDLEVVLR
4	GNDPNATPEKYAK	14	LEGLVSR	24	VFPVDHKAR
5	LDPEPALFVNDYNVER	15	YILVAGR	25	TFTVEK
6	LGDEDIPAYMFKEVAR	16	VFPVDHK	26	DQLRSAMQSR
7	FKHYDVNNEMLHGR	17	DLEVVLR	27	LEGLVSRYAGR
8	DRLGDEDIPAYMFK	18	LGAGAAASVR	28	VGGWISLGAAR
9	YVVEVTTATGKQMLK	19	AVEKDGVR	29	NLNR DQLR
10	LPIPVGVLKPGITYR	20	QVAWLQGR	30	GNVDGDGDFK

**Table 5:** Average degree of polymerization (avDP), content of water-extractable arabinoxylan (WEAX), and arabinoxylan-oligosaccharides (AXOS) content after degradation.

Degradation Time (h)	0	3.5	7	12	24
avDP of WEAX	ND	25.29	21.24	19.12	16.22
Content of WEAX (mg/mL)	1.00	0.98	0.94	0.91	0.90
Free Ara	ND				
Free Xyl	ND				
AXOS2 (μg/mL)	ND	1.38	4.10	4.28	4.63
AXOS3 (μg/mL)	ND	2.96	7.50	10.33	11.51
AXOS4 (μg/mL)	ND	2.57	6.68	9.70	13.87
AXOS5 (μg/mL)	ND	0.31	3.68	5.23	8.74
AXOS6 (μg/mL)	ND	1.48	5.17	9.18	14.46

Note: ND indicates not detected.

**Table 6:** Database retrieval parameters.

Fixed Modifications	Carbamidomethyl (C)
Variable modifications	Oxidation (M)
Enzyme	Trypsin
Maximum Missed Cleavages	1
Peptide Mass Tolerance	20 ppm
Fragment Mass Tolerance	0.6 Da
Mass values	Monoisotopic
Significance threshold	0.05

## References

[1] Mastanjević K., Šarkanj B., Krska R., Sulyok M., Warth B., Mastanjević K., Šarkanj B., Krstanović V. (2018). From malt to wheat beer: A comprehensive multi-toxin screening, transfer assessment and its influence on basic fermentation parameters. Food Chem..

[2] Vietor R.J., Voragen A.G.J., Angelino S.A.G.F. (1993). Composition of non-starch polysaccharides in wort and spent grain from brewing trials with malt from a good malting variety and a feed variety. J. Inst. Brew..

[3] Rakszegi M., Lovegrove A., Balla K., Láng L., Bedő Z., Veisz O., Shewry P.R. (2014). Effect of heat and drought stress on the structure and composition of arabinoxylan and β-glucan in wheat grain. Carbohydr. Polym..

[4] Revanappa S.B., Nandini C.D., Salimath P.V. (2015). Structural variations of arabinoxylans extracted from different wheat (Triticum aestivum) cultivars in relation to chapati-quality. Food Hydrocoll..

[5] Sajib M. (2017). Preparation and Evaluation of Arabinoxylan Based Prebiotics. Master’s Thesis.

[6] Li S.B., Morris C.F., Bettge A.D. (2009). Genotype and environment variation for arabinoxylans in hard winter and spring wheats of the U.S. Pacific Northwest. Cereal Chem..

[7] Guo M.M., Du J.H., Zhang K.L., Jin Y.H. (2014). Content and molecular weight of water-extractable arabinoxylans in wheat malt and wheat malt-based wort with different Kolbach indices. J. Sci. Food Agric..

[8] Mendis M., Simsek S. (2015). Production of structurally diverse wheat arabinoxylan hydrolyzates using combinations of xylanase and arabinofuranosidase. Carbohydr. Polym..

[9] Jin Y.H., Du J.H., Zhang K.L., Xie L., Li P.P. (2012). Relationship between Kolbach Index and other quality parameters of wheat malt. J. Inst. Brew..

[10] Han J.-Y. (2000). Structural characteristics of arabinoxylan in barley, malt, and beer. Food Chem..

[11] Evans D.E., Sheehan M.C., Stewart D.C. (1999). The impact of malt derived proteins on beer foam quality. Part II. The influence of malt foam-positive proteins and non-starch polysaccharides on beer foam quality. J. Inst. Brew..

[12] Mendis M., Simsek S. (2014). Arabinoxylans and human health. Food Hydrocoll..

[13] Song H.Y., Lim H.K., Kim D.R., Lee K.I., Hwang I.T. (2014). A new bi-modular endo-β-1,4-xylanase KRICT PX-3 from whole genome sequence of *Paenibacillus terrae* HPL-003. Enzyme Microb. Technol..

[14] De Souza A.R., de Araújo G.C., Zanphorlin L.M., Ruller R., Franco F.C., Torres F.A., Mertens J.A., Bowman M.J., Gomes E., Da Silva R. (2016). Engineering increased thermostability in the GH-10 endo-1,4-β-xylanase from *Thermoascus aurantiacus* CBMAI 756. Int. J. Biol. Macromol..

[15] Debyser W., Derdelinckx G., Delcour J.A. (1997). Arabinoxylan and arabinoxylan hydrolysing activities in barley malts and worts derived from them. J. Cereal Sci..

[16] Viëtor R.J., Hoffmann R.A., Angelino S.A.G.F., Voragen A.G.J., Kamerling J.P., Vliegenthart J.F.G. (1994). Structures of small oligomers liberated from barley arabinoxylans by endoxylanase from *Aspergillus awamori*. Carbohydr. Res..

[17] Cleemput G., Hessing M., van Oort M., Deconynck M., Delcour J.A. (1997). Purification and Characterization of a β-D-xylosidase and an endo-xylanase from wheat flour. Plant Physiol..

[18] Cleemput G., Van Laere K., Hessing M., Van Leuven F., Torrekens S., Delcour J.A. (1997). Identification and characterization of a nove1 arabinoxylanase from wheat flour. Plant Physiol..

[19] Dornez E., Gebruers K., Delcour J.A., Courtin C.M. (2009). Grain-associated xylanases: Occurrence, variability, and implications for cereal processing. Trends Food Sci. Technol..

[20] Benjavongkulchai E., Spencer M.S. (1986). Purification and characterization of barley-aleurone xylanase. Planta.

[21] Bhardwaj N., Kumar B., Verma P. (2019). A detailed overview of xylanases: An emerging biomolecule for current and future prospective. Bioresour. Bioprocess..

[22] Silva L.A.O., Terrasan C.R.F., Carmona E.C. (2015). Purification and characterization of xylanases from *Trichoderma inhamatum*, Electron. J. Biotechnol..

[23] Xie L., Jin Y.H., Du J.H., Zhang K.L. (2014). Water-soluble protein molecular weight distribution and effects on wheat malt quality during malting. J. Inst. Brew..

[24] Guo X., Jin Y.H., Du J.H. (2017). Extraction and purification of an endo-1,4-β-xylanase from wheat malt. J. Cereal Sci..

[25] Peng Z.J., Jin Y.H., Du J.H. (2019). Enzymatic Properties of endo-1,4-β-xylanase from Wheat Malt. Protein Pept. Lett..

[26] Chai Y.N., Jin Y.H., Du J.H., Li J., Zhang K.L. (2015). Partial characterization of β-D-xylosidase from wheat malts. J. Inst. Brew..

[27] Sozzi G.O., Fraschina A.A., Navarro A.A., Cascone O., Greve L.C., Labavitch J.M. (2002). α-L-Arabino furanosidase activity during development and ripening of normal and ACC synthase antisense tomato fruit. HortScience.

[28] Kong F.J., Oyanagi A., Komatsu S. (2010). Cell wall proteome of wheat roots under flooding stress using gel-based and LC MS/MS-based proteomics approaches. Biochim. Biophys. Acta Biomembr..

[29] Loosveld A.A., Grobet P.J., Delcour J.A. (1997). Contents and structural features of water-extractable arabinogalactan in wheat flour fractions. J. Agric. Food Chem..

[30] Englyst H.N., Cummings J.H. (1984). Simplified method for the measurement of total non-starch polysaccharides by gas-liquid chromatography of constituent sugars as alditol acetates. Analyst.

[31] Courtin C.M., Den Broeck H.V., Delcour J.A. (2000). Determination of reducing end sugar residues in oligo- and polysaccharides by gas-liquid chromatography. J. Chromatogr. A.

[32] Li M.M., Du J.H., Han Y.Y., Li J., Bao J., Zhang K.L. (2019). Non-starch polysaccharides in commercial beers on China market: Mannose polymers content and its correlation with beer physicochemical indices. J. Food Compos. Anal..

[33] Jiang Y., Du J.H., Zhang L.G., Li W.Q. (2018). Properties of pectin extracted from fermented and steeped hawthorn wine pomace: A comparison. Carbohydr. Polym..

[34] Rascón-Chu A., Martínez-Lópe A.L., Carvajal-Millán E., León-Renova N.P., Márquez-Escalante J.A., Romo-Chacón A. (2009). Pectin from low quality ‘Golden Delicious’ apples: Composition and gelling capability. Food Chem..

